# Sex Differences in Intracranial Atherosclerotic Plaques Among Patients With Ischemic Stroke

**DOI:** 10.3389/fcvm.2022.860675

**Published:** 2022-06-30

**Authors:** Xuejiao Yan, Min Tang, Jie Gao, Lihui Wang, Ling Li, Niane Ma, Xiaorui Shi, Xiaoyan Lei, Xiaoling Zhang

**Affiliations:** ^1^Department of MRI, Shaanxi Provincial People's Hospital, Xi'an, China; ^2^Department of Radiology, Xi'an International Medical Center Hospital, Xi'an, China

**Keywords:** atherosclerosis, cardiovascular magnetic resonance imaging, sex differences, ischemic stroke, high-risk plaque

## Abstract

**Objective:**

High-risk intracranial arterial plaques are the most common cause of ischemic stroke and their characteristics vary between male and female patients. However, sex differences in intracranial plaques among symptomatic patients have rarely been discussed. This study aimed to evaluate sex differences in intracranial atherosclerotic plaques among Chinese patients with cerebral ischemia.

**Methods:**

One hundred and ten patients who experienced ischemic events underwent 3T cardiovascular magnetic resonance vessel wall scanning for the evaluation of intracranial atherosclerotic disease. Each plaque was classified according to its likelihood of causing a stroke (as culprit, uncertain, or non-culprit). The outer wall area (OWA) and lumen area of the lesion and reference sites were measured, and the wall and plaque areas, remodeling ratio, and plaque burden (characterized by a normalized wall index) were further calculated. The composition (T_1_ hyperintensity, enhancement) and morphology (surface irregularity) of each plaque were analyzed. Sex differences in intracranial plaque characteristics were compared between male and female patient groups.

**Results:**

Overall, 311 plaques were detected in 110 patients with ischemic stroke (81 and 29 male and female patients, respectively). The OWA (*P* < 0.001) and wall area (*P* < 0.001) of intracranial arterial lesions were significantly larger in male patients. Regarding culprit plaques, the plaque burden in male patients was similar to that in female patients (*P* = 0.178, odds ratio [OR]: 0.168, 95% confidence interval [CI]: −0.020 to 0.107). However, the prevalence of plaque T_1_ hyperintensity was significantly higher than that in female patients (*P* = 0.005, OR: 15.362, 95% CI: 2.280–103.49). In the overall ischemic stroke sample, intracranial T_1_ hyperintensity was associated with male sex (OR: 13.480, 95% CI: 2.444–74.354, *P* = 0.003), systolic blood pressure (OR: 1.019, 95% CI: 1.002–1.036, *P* = 0.031), and current smoker (OR: 3.245, 95% CI: 1.097–9.598, *P* = 0.033).

**Conclusion:**

For patients with ischemic stroke, the intracranial plaque burden in male patients was similar to that in female patients; however, the plaque characteristics in male patients are associated with higher risk, especially in culprit plaques.

## Introduction

Ischemic stroke is a major cause of long-term disability and death (1). Studies on sex differences in the incidence of ischemic stroke have shown that the incidence of major atherosclerotic etiology is lower in women than in men (2), although the overall age-related incidence of stroke is higher in women owing to longer life expectancy (3). The global Burden of Disease, Injury and Risk Factors Study further revealed that the rates of disability-adjusted life year and age-standardized mortality attributable to ischemic stroke were higher in male patients than in female patients among Chinese individuals (4). The exact cause or mechanism underlying this sex-related difference is not fully understood. The results of a 10-year national cohort study on sex-based differences in carotid revascularization showed a low incidence of stroke after carotid endarterectomy (CEA) or carotid artery stent (CAS) in symptomatic male patients, and the functional outcome in male patients was better than that in female patients in subsequent follow-up examinations. These results suggest that male patients may benefit more than female patients after CEA or CAS (5). The study of sex differences in carotid plaques has shown a more stable carotid plaque phenotype in women, which may explain why female patients benefit less from CEA or CAS than do male patients (6, 7). Because artery-to-artery embolization caused by rupture of unstable atherosclerotic plaques is one of the important mechanisms of ischemic stroke, endovascular therapy reduces the incidence of clinical events by removing unstable plaques. Therefore, whether one of the reasons for the differences in ischemic stroke incidence and outcome between male and female patients is related to the sex-specific characteristics of plaques remains to be further studied, especially for the intracranial arteries (including the intracranial part of the internal carotid artery).

To our knowledge, only a few studies have focused on sex differences in arterial plaque characteristics. In a histological analysis of 1,422 patients (453 women, 969 men) who underwent carotid endarterectomy, Vrijenhoek et al. found that among patients with carotid atherosclerotic plaques, the prevalence of plaque intraplaque hemorrhage (IPH) was higher in men than in women (67 vs. 54%; *P* < 0.001). Male secondary cardiovascular disease is associated with local IPH (8). Non-invasive cardiovascular magnetic resonance (CMR) vessel wall imaging has rapidly developed in recent years and it has proven a reliable technique for determining plaque location and identifying plaque high-risk characteristics (9, 10). Large-vessel atherosclerotic disease is associated with high-risk plaque features that strongly suggest plaque instability, including IPH, thin or ruptured fibrous caps, lipid-rich necrotic core (LRNC), positive plaque remodeling, plaque surface irregularity, and plaque enhancement (11–14). By using vessel wall imaging, Zhang et al. found that the prevalence of LRNC (72.3 vs. 46.0%, *P* < 0.05) and IPH (18.6 vs. 4.9%, *P* < 0.05) in symptomatic male patients with carotid plaques was higher than that in their female counterparts (15). Although the Chinese Intracranial Atherosclerosis (CICAS) study (16) suggests that ischemic stroke is mainly caused by intracranial atherosclerosis in the Chinese population, to our knowledge, sex differences in intracranial atherosclerotic plaque characteristics in symptomatic patients have rarely been reported. Voigt et al. assessed intracranial atherosclerotic plaque burden by quantifying plaque calcification on non-contrast computed tomography (NCCT) and found no difference in intracranial plaque burden between male and female patients (17). However, NCCT is not good at identifying other high-risk plaque components and surface morphology, and sex differences in symptomatic patients with intracranial arterial plaque remain unclear. Therefore, the purpose of this study was to comprehensively assess sex differences in intracranial atherosclerotic plaque morphology and high-risk characteristics in patients with ischemic stroke by using CMR vessel wall imaging. A better understanding of sex-specific characteristics of intracranial arterial plaques may contribute to the prevention and management of ischemic stroke.

## Materials and Methods

### Study Population

Between August 2017 and September 2021, 272 patients with transient ischemic attacks or strokes were continuously reviewed using the high-resolution MRI (HR-MRI) database of our medical institution. The patient underwent CMR vessel wall imaging after the onset of intracranial ischemia. The detailed inclusion criteria were as follows: (1) stenosis of at least one intracranial artery, as determined by MR angiography (MRA) or CT angiography (CTA) and (2) presence of one or more traditional risk factors for atherosclerosis, including diabetes mellitus (DM), hyperlipidemia, hypertension, and current smoker. The exclusion criteria were as follows: (1) extracranial carotid artery and bilateral vertebral artery stenosis of >50% or extracranial artery with vulnerable plaque (fibrous cap rupture, LRNC, or IPH); (2) intracranial arterial lumen occlusion; (3) receipt of an intravascular intervention or thrombolytic therapy before CMR vessel wall imaging; (4) ischemic symptoms caused by non-atherosclerotic vascular conditions, such as dissection, vasculitis, Moya Moya disease, and reversible vasoconstriction syndrome; (5) presence of high-risk factors of cardiogenic embolism, such as valvular heart disease, atrial fibrillation, and patent foramen ovale; (6) poor image quality; and (7) a lack of relevant laboratory data for outpatients.

The study protocol was approved by Shaanxi Provincial People's Hospital review board. All study participants provided signed informed consent.

### Clinical Data and Laboratory Measurements

The following clinical characteristics were collected from medical records of all patients: age, sex, smoking status, height, weight, blood pressure, current statin use, and a family history of cardiovascular disease. The laboratory test data collected included triglyceride (TG), total cholesterol (TC), low-density lipoprotein cholesterol (LDL), high-density lipoprotein cholesterol (HDL), apolipoprotein A1, apolipoprotein B, uric acid (UA), homocysteine (Hcy), and glycosylated hemoglobin (HbA1c) levels.

### Definition of Traditional Atherosclerotic Risk Factors

Risk factors included (1) hypertension (mean systolic blood pressure of >130 mmHg and/or mean diastolic blood pressure of >80 mmHg and/or a history of hypertension or use of antihypertensive medication) (18); (2) obesity defined as a body mass index (BMI) of ≥28.0 kg/m^2^ (BMI = weight [kg]/height [m]^2^) (19); (3) dyslipidemia (TC concentration of >5.18 mmol/L and/or TG concentration of >1.7 mmol/L and/or LDL-C concentration of >3.37 mmol/L and/or lipid-lowering drug use); (4) DM (HbA1c level of >6.0% and/or a history of DM and/or hypoglycemic drug use) (20); (5) hyperhomocysteinemia (HHcy; Hcy concentration of ≥20 mmo1/L); and (6) current smoker.

### MRI Protocol

MRI scans were performed using the Philips 3.0T CMR scanner (Ingenia, Philips Medical System, The Netherlands) and a 16-channel head and neck coil. The CMR vessel wall imaging protocol included pre- and post-contrast three-dimensional (3D) volume isotropic turbo spin-echo acquisition (VISTA) and 3D time-of-flight (TOF) MRA. Visualization of vascular stenosis by 3D-TOF MRA was performed with the following parameters: repetition time (TR)/echo time (TE) = 20 ms/3.6 ms; matrix = 256 × 256; field of view (FOV) = 180 × 180 mm^2^; and slice thickness = 5 mm. The scan time was ≈3 min. 3D T_1_-weighted VISTA sequences were obtained for plaque analysis with the following parameters: TR/TE = 700 ms/14 ms; FOV = 80 × 80 mm^2^; slice thickness = 2 mm; layer spacing = 0.5 mm; and matrix = 256 × 256. The scanning time was ~4.5 min. Enhanced images were obtained using repeated T_1_-weighted VISTA sequences after intravenous injection of 0.1 mmol/kg contrast agent (Gadovist®, Bayer Schering Pharma AG, Berlin, Germany) and a delay of ~5 min. Before CMR vessel wall imaging, conventional plain MR scanning was performed, including T_1_-weighted imaging (T_1_WI), T_2_-weighted imaging (T_2_WI), fluid-attenuated inversion recovery, and diffusion-weighted imaging (DWI), to determine the infarct location. The total sequence scanning time was ~20 min. All images were de-identified and digitally stored.

### Image Analysis

**Figure 1:**
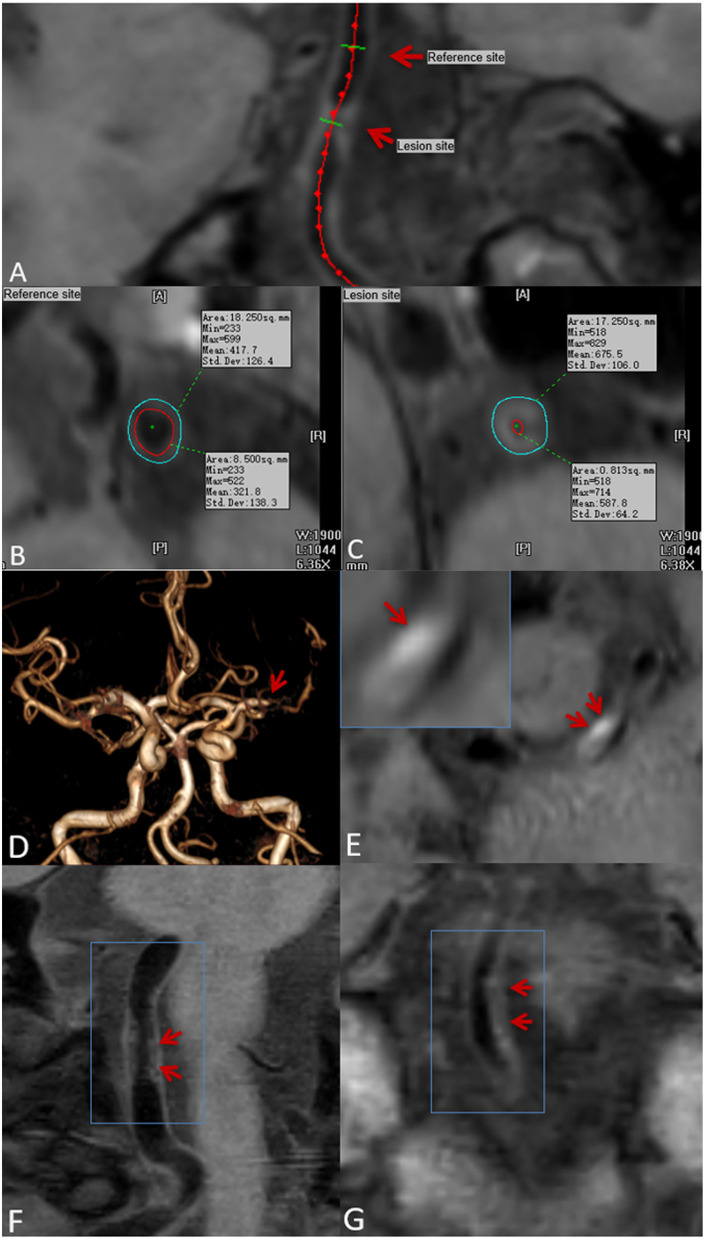
Plaque localization, composition, and surface morphology recognition. Ts-imaging software was used to perform curved planar reconstruction for the vascular axis. **(A)** (red arrow) with the thinnest wall as the reference site **(B)** and the thickest cross-section of the plaque as the lesion site **(C)**. Lumen area (LA) and outer wall area (OWA) were calculated automatically by the software. Time-of-flight magnetic resonance angiography (TOF-MRA) showing stenosis of the M1 segment of the left middle cerebral artery **(D)** (red arrow): pre-contrast CMR vessel wall imaging shows an eccentric plaque at the stenosis, and a high-signal shadow can be seen in the plaque **(E)** (red arrow), indicating intraplaque hemorrhage. Plaque surface irregularity **(F)**, and regularity **(G)** (red arrow).

All CMR vessel wall imaging data were transformed using semi-automatic software (tsimaging.net) and RadiAnt DICOM Viewer (Version 2020.2. https://www.radiantviewer.com) for analysis. According to the overall signal-to-noise ratio and the clarity of the vessel wall boundary, the image quality score was determined using the four-point Likert scale (1, poor; 2, edge; 3, good; and 4, excellent). Images with a quality score of 1 were excluded (11). Two experienced CMR vessel wall imaging reviewers (6 and 4 years of experience in plaque imaging, respectively) who were blinded to clinical information and routine brain imaging, independently analyzed all the plaques in the patients' intracranial arteries, including bilateral internal carotid arteries C6-7, middle cerebral artery M1-2, anterior cerebral artery A1-2, vertebral artery V4, posterior cerebral artery P1-2, and basilar artery. For each plaque, the entire vascular segment containing the plaque was analyzed. A curved reconstruction of the vascular axis was performed using TS*-*imaging Software, with a 2.0-mm-thick section of the vertical vascular axis to reconstruct the vessel cross-section. For each axis, a cross-section with the thickest plaques was selected as the lesion site. The cross-section containing the thinnest wall was selected as the reference point (Figures 1A–C). The reference point was located at the proximal end of the plaque site (i.e., the nearest plaque-free segment proximal to the lesion site). If a proximal reference site was not available, then the neighboring distal site was used instead. TS imaging software was further used to delineate lumen and outer wall profiles were delineated at lesion and reference sites, and quantitative MRI measurements were automatically generated, including lumen area (LA) and outer wall area (OWA). Wall area (WA) was calculated using the following formula WA = OWA—LA. Normalized wall index (NWI) was used to characterize plaque burden (21) and calculated as follows:


$$\begin{array}{l} \text{NWI}=\frac{\text{OWA}-\text{LA}}{\text{OWA}}\;\times \;100\%. \end{array}$$

**Figure 2:**
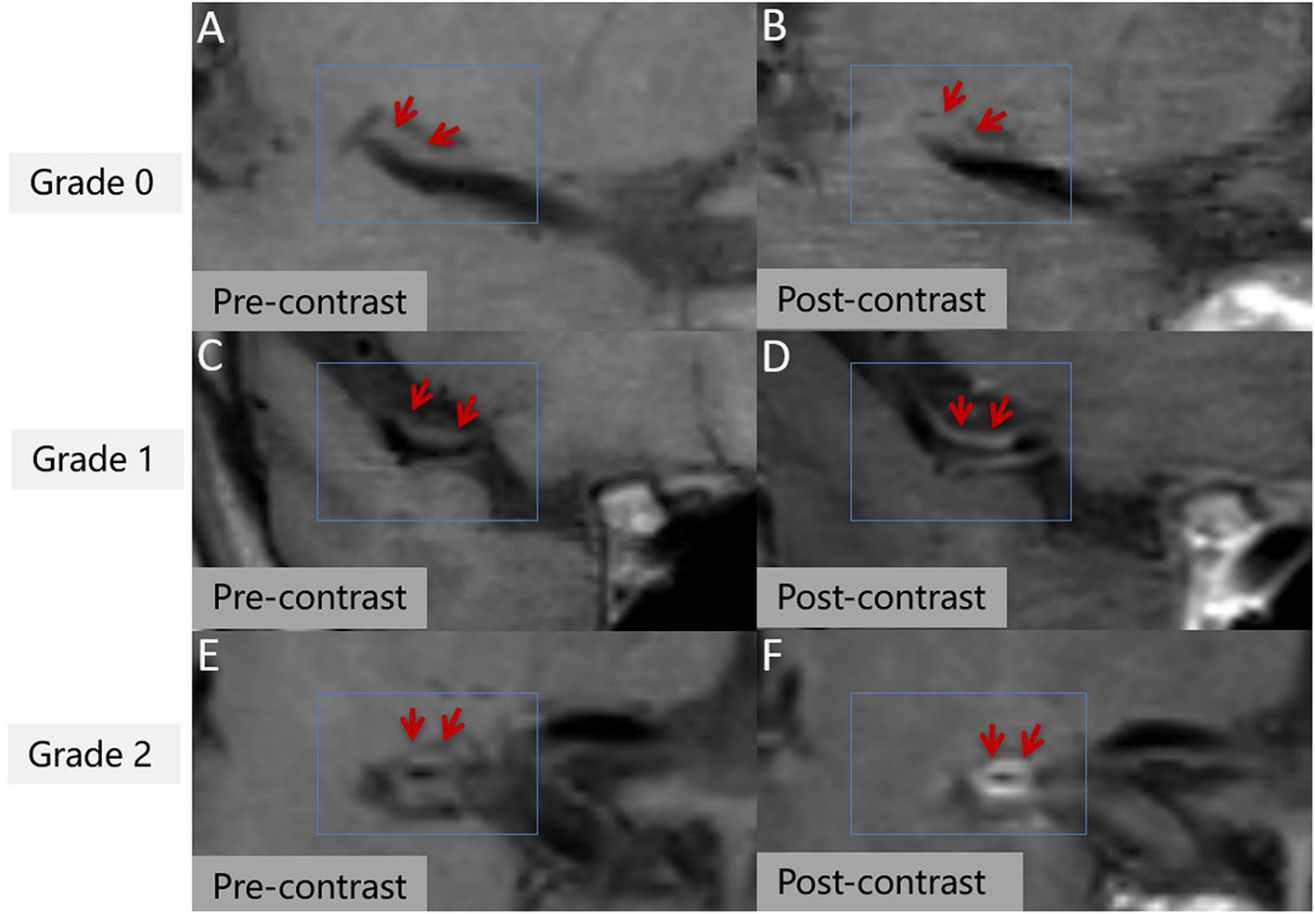
Intracranial plaque enhancement at different levels pre- **(A,C,E)** and post-contrast **(B,D,F)**. 3D T_1_ volume isotropic turbo spin-echo acquisition (T1 VISTA) image showing eccentric atherosclerotic plaques (red arrow). Grade 0 enhancement **(B)**, grade 1 enhancement **(D)**, grade 2 enhancement **(F)**.

Stenosis rate was defined as (1 – lesion LA/reference LA) × 100% (22). The mean values measured by the two reviewers was used for the subsequent analysis. With regard to T_1_ hyperintensity components, a high plaque signal was detected on T_1_WI, which was considered positive for intraplaque hemorrhage (23). A high signal intensity was defined as an area with an adjacent muscle signal intensity of >150% (Figures 1D,E) (24). The remodeling ratio (RR) was defined as OWA at plaque/OWA at reference. Three remodeling categories were defined as previously described: positive (outward expansion of the wall), RR > 1.05; intermediate, 0.95 ≤ RR ≤ 1.05; and negative (vessel wall shrinkage), RR < 0.95 (25). Regarding plaque surface morphology, an irregularity was defined as discontinuity at the plaque juxta luminal surface (Figure 1F) or smooth regularity at the plaque inner wall (Figure 1G) (22). Intracranial plaque enhancement was classified into three grades on post-contrast T_1_-VISTA images: grade 0, no enhancement, defined as the degree of enhancement similar to that of normal vessel wall; grade 1, defined as the degree of enhancement less than that of the pituitary infundibulum but greater than that of normal vessel wall; and grade 2, defined as the degree of enhancement similar to or greater than that of the pituitary infundibulum (Figure 2). For inconsistent cases, another senior neuroradiologist (with 10 years of experience in imaging-based diagnosis) reassessed images and assisted in reaching a consensus.

### Plaque Classification

**Figure 3:**
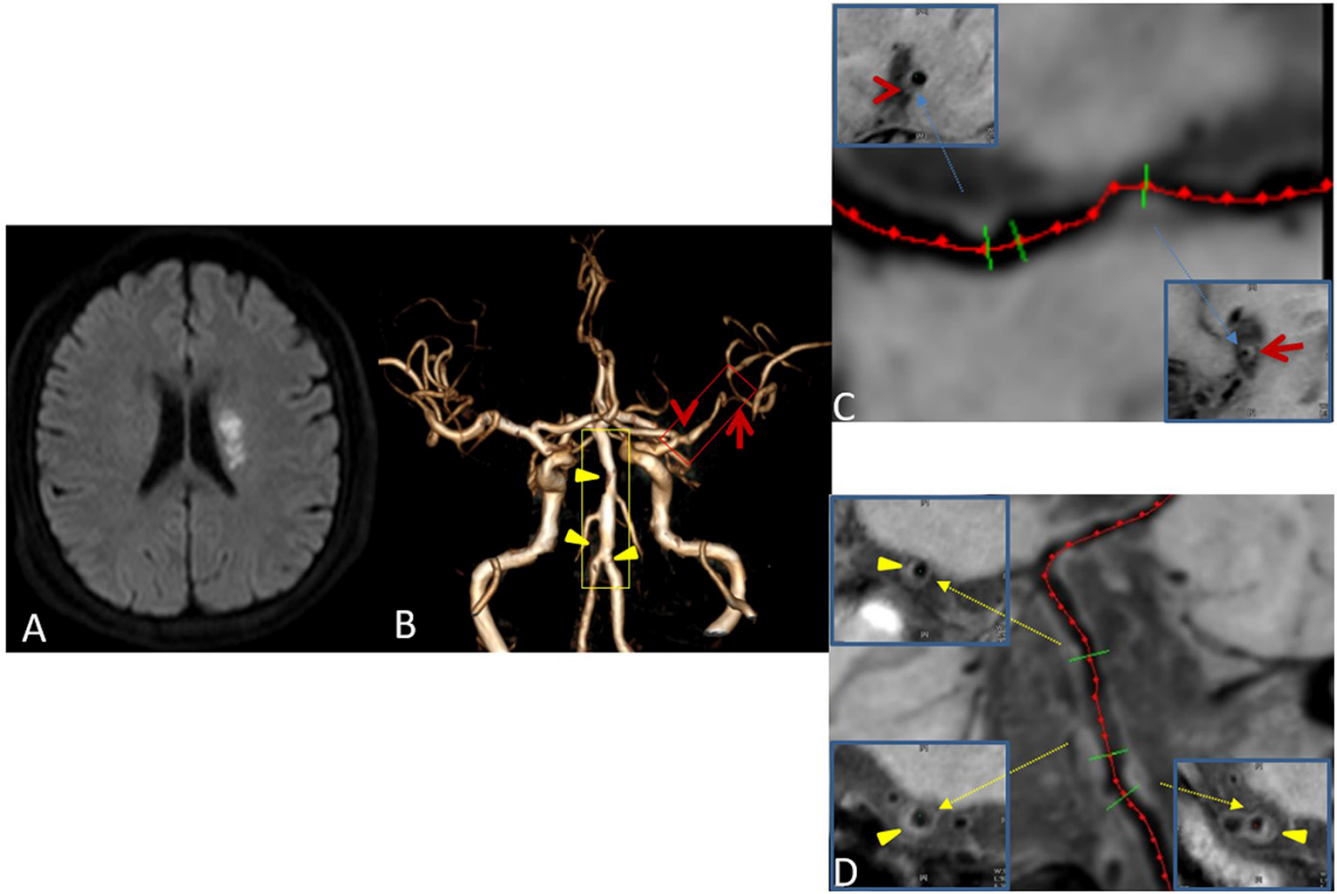
Plaque Classification. Male, 42 years old, presenting with sudden right limb weakness for 8 h. Diffusion-weighted imaging (DWI) shows acute left lateral paraventricular infarction **(A)**. Time-of-flight magnetic resonance angiography (TOF MRA) shows stenosis at two sites in the M1 segment of the left middle cerebral artery (red box) and three sites in the basilar and left vertebral arteries (yellow box) **(B)**. Curved planar reconstruction images show severe stenosis at the distal end of M1 as the culprit plaque (red arrow), and mild stenosis at the proximal end of M1 as an uncertain plaque (red Arrowhead) **(C)**. The three plaques of the basilar artery and left vertebral artery are non-culprit plaques (Yellow triangular arrowheads) **(D)**.

On the basis of the possibility of stroke identified on routine MRI, each plaque was categorized as culprit, uncertain, or non-culprit. If a plaque was the only lesion in the vascular area of the stroke or if it was the narrowest lesion among multiple plaques in the same vascular area of the middle wind, it was categorized as a culprit plaque. If a plaque was not the narrowest lesion in the same vascular area of the stroke, it was categorized as uncertain. If a lesion was not in the vascular area of the stroke, it was categorized as non-culprit (Figure 3). For patients with transient ischemic attack (TIA), plaque classification should be performed if the symptoms can be localized to the corresponding vascular region (21).

### Reproducibility Assessment

In this study cohort, 20 patients were randomly selected, and two reviewers performed all measurements independently to determine the reliability between observers.

### Statistical Analysis

The Shapiro-Wilk test was used to identify data with a normal distribution. In univariable analysis, normally distributed data are presented as mean ± standard deviation and did not conform to M [P_25_ P_75_]. Classified data are represented by counts. The clinical characteristics and plaque characteristics of male and female patients were compared using independent sample *t*-test, Mann–Whitney *U*-test, or chi-square test as appropriate. Model 1 and model 2 were used to check the sex differences in the size and composition of culprit plaques. In model 1, multivariate linear regression was used to model continuous variables, and logistic regression was used to evaluate binary variables. Sex-related baseline clinical indicators (*P* < 0.1) or variables reported in the literature that might contribute to the effect were considered confounding variables and were included in the multivariate analysis. NWI was added as a confounding variable in model 2. The results were presented by the regression slope (β) or odds ratio (OR) and the corresponding 95% confidence interval (CI). In the overall sample, multivariate logistic regression was performed to assess the relationship between sex and plaque characteristics (Model 3). All statistical tests were two-sided, and *P* < 0.05 was considered statistically significant. Inter-reader agreement was estimated based on repeat readings of all detected lesions from two readers. The reproducibility of plaque features (including plaque enhancement, surface irregularity, and T_1_ hyperintensity) was estimated using Cohen's Kappa statistic, and the intra-group correlation coefficient was used to calculate the repeatability of the plaque measurement data. All statistical analyses were performed using SPSS version 20 (SPSS Inc., Chicago, IL, USA).

## Results

### Patient Characteristics

A total of 272 subjects were recruited in this study, of which 162 were excluded due to the following reasons: (1) carotid artery origin or intracranial artery occlusion, 66 patients; (2) thrombolysis, 18 patients; (3) dissection, 21 patients; aneurysms, 22 patients; vasculitis, 5 patients; Moyamoya disease, 14 patients; (5) suspected cardiogenic infarction, 6 patients; (4) no clinical laboratory test data, 6 outpatients; and 5) poor image quality, 4 patients. Supplementary Figure S1 shows the flowchart of patient recruitment. A total of 110 patients finally met all the inclusion and exclusion criteria.

Among these 110 patients, 81 were males (55.42 ± 14.26 years) and 29 were females (62.07 ± 12.15 years). The clinical characteristics of the study population are shown in Table 1. In comparison with females, males presented with cerebral ischemic symptoms at a younger age (*P* = 0.027) and had higher levels of Hcy (*P* = 0.007) and UA (*P* = 0.010). Males showed a significantly higher prevalence of current smoking (51.8 vs. 6.8%, *P* < 0.001) and HHcy (48.1 vs. 20.7%, *P* = 0.010) than female. However, males showed lower HDL (1.02 ± 0.23 vs. 1.21 ± 0.33, *P* = 0.006) and apolipoprotein A1 (1.18 ± 0.22 vs. 1.32 ± 0.26, *P* = 0.005) levels than females. Plaque quantity and plaque distribution showed no significant difference between male and female patients (Table 1).

**Table 1:** Differences in clinical data and plaque distribution between male and female patients.

**Characteristics**	**All patients (*N =* 110)**	**Male patients (*N =* 81)**	**Female patients (*N =* 29)**	***P***
Age (years)	57.17 ± 14.00	55.42 ± 14.26	62.07 ± 12.15	0.027
BMI (kg/m^2^)	24.9 (22.6, 27.0)	25.0 (22.9, 27.5)	23.90 ± 3.46	0.143
**Clinical findings**				
Systolic blood pressure (mmHg)	153.82 ± 30.32	151.90 ± 29.93	159.17 ± 31.29	0.270
Diastolic blood pressure (mmHg)	89.87 ± 17.69	90.69 ± 19.01	87.59 ± 13.39	0.420
History of CHD/heart failure	9 (8.1%)	6 (7.4%)	3 (10.3%)	0.696
Current stain use	77 (70%)	58 (71.6%)	19 (65.5%)	0.638
**Laboratory findings**				
TC (mmol/L)	4.0 (3.22, 4.67)	4.11 ± 1.34	4.06 (3.19, 4.68)	0.541
TG (mmol/L)	1.37 (1.01, 1.81)	1.37 (1.02, 1.92)	1.35 (0.99, 1.73)	0.699
LDL-C (mmol/L)	2.27 (1.80, 2.83)	2.48 ± 1.14	2.19 (1.82, 2.59)	0.500
HDL-C (mmol/L)	1.07 ± 0.27	1.02 ± 0.23	1.21 ± 0.33	0.006
Apo A1	1.22 ± 0.23	1.18 ± 0.22	1.32 ± 0.26	0.005
Apo B	0.83 ± 0.28	0.84 ± 0.30	0.79 ± 0.25	0.522
HbA1c %	5.80 (5.50, 6.33)	5.70 (5.50, 6.35)	6.0 (5.55, 6.35)	0.323
Hcy (μmol/L)	16.46 (14.0, 23.7)	17.7 (15.0, 26.4)	15.0 (11.6, 17.9)	0.007
UA (mmol/L)	333.72 ± 95.10	350.00 ± 94.87	290.21 ± 82.66	0.010
**Vascular risk factors *N* (%)**				
Current smoker	44 (40%)	42 (51.8%)	2 (6.8%)	<0.001
History of HT	80 (72.7%)	58 (71.6%)	22 (75.8%)	0.809
History of DM	45 (40.9%)	31 (38.3%)	14 (48.3%)	0.384
Dyslipidemia	45 (40.9%)	32 (39.5%)	13 (44.8%)	0.663
Hyperuricemia	21 (19.1%)	15 (18.5%)	6 (20.7%)	0.799
HHcy	45 (40.9%)	39 (48.1%)	6 (20.7%)	0.010
Obesity	17 (15.5%)	13 (16.0%)	4 (13.7%)	1.000
Stroke				0.751
Acute	74	53	21	
Subactue	4	3	1	
Chronic	10	9	1	
Transient ischemic attack	22	16	6	
Plaque characteristics, *N* (%)^*^				
Number of plaques per patient				0.676
1	30	19	11	
2	25	19	6	
3	20	16	4	
4	15	11	4	
≥5	20	16	4	
Plaque number	3 (1,4)	3 (2,4)	2 (1,4)	0.218
Plaque distribution				0.468
Anterior circulation	39	26	13	
Posterior circulation	12	10	2	
Both	59	45	14	
Vessel segment, Number of plaques per segment (%)^†^				0.786
Anterior cerebral artery	7	6	1	
Internal carotid artery	65	51	14	
Middle cerebral artery	105	75	30	
Basilar artery	56	44	12	
Posterior cerebral artery	9	6	3	
Vertebral artery	69	54	15	

*BMI, body mass index; TC, total cholesterol; TG, triglyceride; LDL-C, low-density lipoprotein cholesterol; HDL-C, high density lipoprotein cholesterol; Apo, Apolipoprotein; HT, hypertension; DM, diabetes mellitus; UA, uric acid; CHD, coronary heart disease; HHcy, hyperhomocysteinemia*

* *Percentages based on patients;*

† *Percentages based on plaques*.

### Comparison of Plaque Characteristics Between Male and Female Patients

A total of 311 plaques were detected in 110 patients. In assessments performed with all plaque samples, OWA (16.2 [11.8, 21.7] mm^2^ vs. 11.6 [8.5, 16.8] mm^2^, *P* < 0.001), WA (13.2 [9.4, 17.4] mm^2^ vs. 8.4 [7.1, 11.8] mm^2^, *P* < 0.001), plaque area (3.3 [1.8, 5.7] mm^2^ vs. 2.1 [0.9, 4.2] mm^2^, *P* = 0.001) and RR (0.99 [0.89, 1.11] vs. 0.94 [0.83, 1.06], *P* = 0.046) in male patients were significantly higher than those in female subjects. However, the two groups showed no significant differences in lumen stenosis (*P* = 0.422) and NWI (*P* = 0.081). In addition, male and female patients showed no significant differences when stratified by the type of remodeling. In comparisons based on plaque composition and morphology, we found a significantly higher prevalence of T_1_ hyperintensity in male plaques than in female plaques (Table 2). Sex differences were also observed in intracranial artery reference site measurements, as shown in Supplementary Table S1.

**Table 2:** Sex differences in intracranial plaque characteristics.

**Characterastics**	**All plaque (*n =* 311)**			**Culprit plaque (*n =* 97)**			**Uncertain plaque (*n =* 64)**			**Non-culprit plaque (*n =* 150)**		
	**Male (*n =* 236)**	**Female (*n =* 75)**	***P***	**Male (*n =* 71)**	**Female (*n =* 26)**	***P***	**Male (*n =* 52)**	**Female (*n =* 12)**	***P***	**Male (*n =* 113)**	**Female (*n =* 37)**	***P***
Outer wall area (mm^2^)	16.2 (11.8, 21.7)	11.6 (8.5, 16.8)	<0.001	13.97 ± 5.14	9.02 (7.14, 10.66)	<0.001	16.8 (12.1, 23.4)	15.60 ± 5.78	0.449	18.4 (13.9, 23.6)	12.3 (10.9, 18.5)	<0.001
Lumen area (mm^2^)	2.8 (0.7, 5.2)	3.1 (0.9, 4.5)	0.422	0.72 (0.37, 2.39)	1.19 (0.39, 2.79)	0.748	1.67 (0.69, 5.29)	3.28 (0.99, 5.08)	0.409	4.5 (2.7, 6.4)	3.56 ± 1.88	0.024
Wall area (mm^2^)	13.2 (9.4, 17.4)	8.4 (7.1, 11.8)	<0.001	12.40 ± 4.82	7.17 (5.69, 8.46)	<0.001	14.92 ± 5.93	11.95 ± 4.80	0.082	13.5 (9.4, 17.8)	10.28 ± 3.19	<0.001
NWI (%)	81.5 (71.6, 93.3)	80.5 (67.8, 90.2)	0.081	92.3 (83.9, 97.1)	86.8 (74.3, 94.5)	0.032	86.0 (72.7, 94.8)	78.36 ± 14.56	0.139	75.08 ± 10.98	75.07 ± 11.04	0.996
Plaque area (mm^2^)	3.3 (1.8, 5.7)	2.1 (0.9, 4.2)	0.001	3.22 (1.57, 4.91)	2.09 (1.25, 3.93)	0.222	4.80 ± 3.99	3.23 ± 3.05	0.209	3.23 (1.77, 6.6)	2.0 (0.9, 3.3)	0.003
Remodeling ratio	0.99 (0.89, 1.11)	0.94 (0.83, 1.06)	0.046	0.96 ± 0.21	0.91 (0.79, 0.98)	0.290	1.09 ± 0.32	0.98 ± 0.31	0.282	1.0 (0.91, 1.14)	0.99 ± 0.18	0.304
Stenosis%	56.4 (33.9, 82.5)	49.0 (31.3, 82.2)	0.332	82.6 (61.9, 92.1)	75.3 (44.0, 89.3)	0.222	57.80 ± 28.88	51.09 ± 31.39	0.477	43.93 ± 23.04	42.40 ± 25.64	0.733
Remodeling category			0.355			0.469			0.706			0.601
Positive, *N* (%)	86 (36.4%)	19 (25.3%)		20 (28.2%)	4 (15.4%)		22 (42.3%)	4 (33.3%)		44 (38.9%)	11 (29.7%)	
Intermediate, *N* (%)	53 (22.5%)	18 (24%)		13 (18.3%)	5 (19.2%)		11 (21.2%)	2 (16.7%)		29 (25.7%)	11 (29.7%)	
Negative, *N* (%)	97 (41.1%)	38 (50.7%)		38 (53.5%)	17 (65.4%)		19 (36.5%)	6 (50%)		40 (35.4%)	15 (40.5%)	
Plaque enhancement			0.948			1.000			0.707			0.896
Grade 0, *N* (%)	77 (32.6%)	26 (34.6%)		11 (15.5%)	4 (15.3%)		10 (19.2%)	3 (25%)		56 (49.6%)	19 (51.4%)	
Grade 1, *N* (%)	75 (31.8%)	23 (30.8%)		19 (26.7%)	7 (26.9%)		18 (34.6%)	5 (41.6%)		38 (33.6%)	11 (29.7%)	
Grade 2, *N* (%)	84 (35.6%)	26 (34.6%)		41 (57.8%)	15 (57.7%)		25 (48.1%)	4 (33.3%)		19 (16.8%)	7 (18.9%)	
T_1_ hyperintensity *N* (%)	34 (14.4%)	3 (4%)	0.014	24 (33.8%)	2 (7.6%)	0.010	7 (13.5%)	0 (0%)	0.331	3 (2.7%)	1 (2.7%)	1.000
Surface irregularity *N* (%)	47 (19.9%)	9 (12%)	0.126	21 (28.2%)	3 (11.5%)	0.109	15 (28.8%)	4 (33.3%)	0.739	12 (10.6%)	2 (5.4%)	0.519

In culprit plaques, OWA (13.97 ± 5.14 vs. 9.02 [7.14, 10.66], *P* < 0.001) and WA (12.40 ± 4.82 vs. 7.17 [5.69, 8.46], *P* < 0.001) were significantly higher in male patients than in female patients. Plaque NWI was also higher in male patients (92.3 [83.9, 97.1] vs. 86.8 [74.3, 94.5], *P* = 0.032). In terms of plaque composition and morphology, the prevalence of T_1_ hyperintensity in male patients was significantly higher than that in female patients (Table 2). In model 1, after adjusting for confounding factors, including age, BMI, systolic blood pressure, HDL level, apolipoprotein A1 level, UA level, current smoker, HHcy level, and hyperuricemia, OWA and WA still showed significant differences between male and female patients, while NWI showed no statistical difference (OR: 0.168, 95% CI: −0.020 to 0.107, *P* = 0.178). In addition, no significant difference was observed in plaque surface irregularity in univariate analysis. However, in model 1, after adjusting for clinical confounders, marginal statistical differences were achieved (OR: 5.176, 95% CI: 1.046–25.60, *P* = 0.044). After further adjustment for clinical confounders and NWI (model 2), plaque T_1_ hyperintensity remained higher in male patients than in female patients (OR: 15.36, 95% CI: 2.280–103.5, *P* = 0.005) (Table 3).

**Table 3:** Univariate comparison and multivariate regression models of gender differences in Culprit plaque characteristics.

**Variable**	**Mean ±SD or M [P_25_, P_75_] or n (%)**			**Adjusted for clinical factors^a^**			**Adjusted for NWI^b^**		
	**Male (*n =* 71)**	**Female (*n =* 26)**	***P***	**β or OR^c^**	**(95% CI)**	***P***	**β or OR^c^**	**(95% CI)**	***p***
Outer wall area (mm^2^)	13.97 ± 5.14	9.02 (7.14, 10.66)	<0.001	0.545	3.634–8.975	<0.001	—	—	—
Lumen area (mm^2^)	0.72 (0.37, 2.39)	1.19 (0.39, 2.79)	0.748	0.023	−0.849 to 1.024	0.853	—	—	—
Wall area (mm^2^)	12.40 ± 4.82	7.17 (5.69, 8.46)	<0.001	0.571	3.760–8.675	<0.001	—	—	—
NWI (%)	92.3 (83.9, 97.1)	86.8 (74.3, 94.5)	0.032	0.168	−0.020 to 0.107	0.178	—	—	—
Plaque area (mm^2^)	3.22 (1.57, 4.91)	2.09 (1.25, 3.93)	0.222	0.156	−0.641 to 2.591	0.234	—	—	—
RR	0.96 ± 0.21	0.91(0.79,0.98)	0.290	−0.015	−0.147 to 0.131	0.912	—	—	—
Stenosis>50%	59 (83.1%)	19 (73.1%)	0.271	2.100	0.436–10.11	0.355	0.210	0.002–29.16	0.535
Positive remodeling	20 (28.2%)	4 (15.4%)	0.289	2.205	0.481–10.11	0.308	2.126	0.458–9.880	0.336
Plaque enhancement	60 (84.5%)	22 (84.6%)	1.000	0.576	0.098–3.379	0.541	0.238	0.024–2.370	0.221
T_1_ hyperintensity	24 (33.8%)	2 (7.6%)	0.010	18.991	2.884–125.05	0.002	15.36	2.280–103.5	0.005
Surface irregularity	21 (28.2%)	3 (11.5%)	0.109	5.176	1.046–25.60	0.044	4.365	0.861–22.14	0.075

a *Model 1: adjusted for age, BMI, systolic blood pressure, HDL, Apolipoprotein A1, UA, current smoker, and HHcy*.

b *Model 2: further adjusted for all factors in model 1, and additionally NWI*.

c *Value is linear regression slope β for continuous measurements or logistic regression OR for binary measurements*.

*RR, remodeling ratio; NWI, Normalized wall index*.

Uncertain plaques showed no significant differences in plaque measurements, plaque composition, and morphology between male and female patients. In non-culprit plaques, the OWA, LA, WA, and plaque area of male patients were larger than that of female patients, while there were no significant differences in the other plaque characteristics (Table 2).

### Association Between Sex and Plaque Characteristics

Binary logistic regression analysis (Model 3) was used to analyze the correlation between sex and the presence of T_1_ hyperintensity in patients. Male sex (OR: 13.480, 95% CI: 2.444–74.354, *P* = 0.003), systolic blood pressure (OR: 1.019, 95% CI: 1.002–1.036, *P* = 0.031), and current smoker (OR: 3.245, 95% CI: 1.097–9.598, *P* = 0.033) were associated with T_1_ hyperintensity (Table 4).

**Table 4:** Multivariate analysis identified factors related to intracranial T_1_ hyperintensity^a^.

**Variable**	**B**	**OR**	**95% CI**	***P***
Age (years)	−0.031	0.969	0.933-1.007	0.110
BMI (kg/m^2^)	−0.031	0.969	0.866–1.084	0.585
Sex	2.601	13.480	2.444–74.354	0.003
HDL–C (mmol/L)	1.026	2.791	0.160–48.690	0.482
Apolipoprotein A1	−2.549	0.078	0.003–2.138	0.131
Systolic blood pressure (mmHg)	0.019	1.019	1.002–1.036	0.031
HHcy^b^	0.927	2.527	0.883–7.235	0.084
Hyperuricemia^c^	0.436	1.547	0.426–5.621	0.508
Current smoker	1.177	3.245	1.097–9.598	0.033

a *Multivariate analysis is Model 3*;

b *HHcy>20 μmol/L*;

c *Hyperuricemia: male> 416 μmol/L, female> 357 μmo1/L*.

### CMR Vessel Wall Imaging Measurement Reproducibility

Table 5 summarizes the inter-observer reproducibility data. All measurements showed excellent inter-observer agreement (intra-class correlation coefficient and Cohen's Kappa >0.80).

**Table 5:** Inter-observer reproducibility (*N* = 20).

**Parameters**	**Inter-observer**	
	**Intra-class correlation coefficients/Cohen's kappa**	**95%CI**
Plaque number	0.863	0.686–0.943
T_1_ hyperintensity	0.901	
Enhancement grade	0.849	
Surface irregularity	0.871	
**Lesion site**		
Outer wall area	0.921	0.846–0.960
Lumen area	0.880	0.770–0.939
**Reference site**		
Outer wall area	0.901	0.810–0.950
Lumen area	0.862	0.738–0.929

## Discussion

Studies on cerebrovascular disease have identified sex-related differences in baseline characteristics, symptoms, etiology, and outcome between male and female patients (26–29). However, the pathophysiological mechanisms underlying these differences remain unclear. Differences in the stability of atherosclerotic plaques may be one explanation for these findings. In this study, we used three-dimensional multi-contrast CMR vessel wall imaging to explore sex differences in symptomatic intracranial atherosclerotic plaques. The main findings are as follows: (1) the outer wall area and wall area of intracranial artery lesions in male patients with ischemic stroke were significantly larger than those in female patients. (2) Among the culprit plaques causing symptoms, the plaque burden in male patients was similar to that in female patients, but the T_1_ hyperintensity in plaques in male patients was significantly higher than that in female patients. (3) In patients with ischemic stroke, T_1_ hyperintensity is associated with male sex, systolic blood pressure, and current smoker. These findings suggest that the risk of intracranial arterial plaques is higher in symptomatic men than in women, particularly for culprit plaques. Therefore, sex-related differences should receive attention in the management and prevention of ischemic stroke.

Our study, which included all intracranial arteries in symptomatic patients, showed significantly greater intracranial vascular outer wall area and wall area in male patients than in female participants, especially in the culprit plaque, even after adjusting for confounding factors. This is similar to the findings for other blood vessels, including the carotid artery (30–32). Krejza et al. found that the diameter of the common carotid artery and internal carotid artery was significantly smaller in women than in men, even after adjusting for age and blood pressure (31). In addition, we found that the outer wall area and vessel wall area in the reference sites of the intracranial artery were larger in male patients than in female patients (Supplementary Table S1). Cogswell et al. used 3T intracranial vessel wall imaging to measure the intracranial artery size in healthy individuals, and the results showed that lumen diameter and the outer wall diameter of the intracranial artery in normal males were larger than those in females (*P* < 0.05). The normal intracranial artery size has been already suggested to show sex differences (33). However, no significant difference was observed in NWI between male and female patients after standardizing vascular area, especially in culprit plaques. Although the plaque burden (with NWI as an indicator) differed between male and female patients before adjustment, after adjusting for clinical risk factors, no significant difference was observed between male and female patients. Thus, men and women will have similar plaque burden under comorbid risk factors, even if their vessels are different in size.

The T_1_ hyperintensity in intracranial arterial plaques has been identified histologically as intraplaque hemorrhage (23, 34). In our subgroup analysis, we found that T_1_ hyperintensity in culprit plaques was more prevalent in males than in females. This is consistent with the findings for the carotid arteries, with male patients showing a higher incidence of carotid IPH on the symptomatic side (15). Singh et al. found less carotid IPH in women before age 65, while the risk of carotid IPH was similar in women and men as postmenopausal age increased. Thus, estradiol may play a role in IPH (35). The mean age of female patients in this study was 62 years, which may also explain the lower incidence of IPH in female patients. T_1_ hyperintensity-associated IPH has been shown to be a very important predictor of arterial plaque vulnerability and is associated with rapid progression of symptomatic atherosclerotic plaque (23, 36). Studies on sex hormones and plaque risk have shown that estradiol improves vascular endothelial function and reduces the progression of atherosclerosis. Mercuro et al. further reported an increased susceptibility to cardiovascular disease (CVD) after postmenopausal estrogen failure (37). However, the mechanisms underlying age-specific sex differences in intracranial arterial plaques high-risk characteristics may need to be explored prospectively. For culprit plaques, male patients also showed a higher prevalence of plaque surface irregularities than female patients, achieving marginal significance in a multivariate analysis (model 1) that was adjusted for potential clinical confounders. The coexistence of vulnerable plaque features such as intraplaque hemorrhage and plaque surface irregularities may be due to plaque margin rupture that increases the risk of surface thrombosis (38). Therefore, we found that symptomatic males have a higher risk of intracranial arterial plaque, particularly in culprit plaques, which may help explain the sex differences in stroke incidence and outcome. Moreover, in the overall sample, we found that the presence of T_1_ hyperintensity was independently associated with male sex, systolic blood pressure, and current smoker. Male patients in this sample had a significantly higher smoking rate, and nicotine can accelerate plaque growth and is associated with increased angiogenesis within plaques (39). The underlying pathophysiological mechanism associated with systolic blood pressure and IPH may be that systolic blood pressure enhances plaque microvasculogenesis, thereby increasing the risk of IPH (40).

Zhang et al. (15) found that the incidence of IPH in asymptomatic lateral carotid arteries was also higher in males than in females, while in our study, there was no significant difference in T_1_ hyperintensity in uncertain plaques and non-culprit plaques between male and female patients. This inconsistency may be attributable to the following reasons: (1) Zhang's study included patients with acute ischemic stroke who developed symptoms within 2 weeks, so atherosclerotic lesions may be more severe. In contrast, our study included patients with ischemic stroke (acute, subacute, and chronic) and TIA. (2) Zhang's study evaluated carotid artery plaques, while this study assessed intracranial anterior and posterior circulation plaques. Plaque characteristics may differ depending on the lesion site. In the non-culprit plaque group, we found that the plaque area in male patients was larger than that in female patients with similar stenosis. Plaque size can be used as a risk predictor of clinical outcomes (41), so more attention needs to be paid to non-culprit plaques in the monitoring, screening, and stroke prevention of intracranial atherosclerosis in the Chinese population, especially in men.

This study also had some noteworthy limitations. First, this was a retrospective cross-sectional study, and the direct correlation between sex differences in intracranial plaque and subsequent stroke risk cannot be elucidated. Therefore, prospective studies should be designed to assess the causal relationship between sex differences in intracranial plaques and the subsequent clinical events. Second, this study was conducted using data of a single center, so the results need to be verified in a multi-center investigation. Finally, due to the small number of female patients in the sample and the lack of relevant clinical information, stratification according to menopause could not be performed. In future studies, we aim to increase the number of participants and collect relevant clinical data for further analysis.

In conclusion, our findings highlight the sex-specific characteristics of intracranial atherosclerotic plaques. The results suggest that men with ischemic stroke, while having a similar intracranial plaque burden to women, have a higher plaque risk profile, especially for culprit plaques. multi-contrast CMR vessel wall imaging can help stroke patients of different sexes better understand their atherosclerosis risk and further optimize their clinical management.

## Data Availability Statement

The original contributions presented in the study are included in the article/Supplementary Material, further inquiries can be directed to the corresponding author/s.

## Ethics Statement

The studies involving human participants were reviewed and approved by Shaanxi Provincial People's Hospital. The patients/participants provided their written informed consent to participate in this study.

## Author Contributions

XY conducted data collection, statistical analysis, data interpretation, and manuscript writing. NM, MT, and XS review the images. XL and LW provided assistance in data collection. LL contributed to the statistical analysis. JG and XZ contributed to research design, data interpretation, and manuscript revision. All authors contributed to the article and approved the submitted version.

## Funding

This research was supported by the National Natural Science Foundation of China (81270416), the Key Research and Development Program of Shaanxi Province of China (2018ZDXM-SF-038), and the Social Development Science and Technology Research Project of Shaanxi Province of China (2021SF-064).

## Conflict of Interest

The authors declare that the research was conducted in the absence of any commercial or financial relationships that could be construed as a potential conflict of interest.

## Publisher's Note

All claims expressed in this article are solely those of the authors and do not necessarily represent those of their affiliated organizations, or those of the publisher, the editors and the reviewers. Any product that may be evaluated in this article, or claim that may be made by its manufacturer, is not guaranteed or endorsed by the publisher.

## Supplementary Material

Supplementary Figure S1 Flow chart of patient recruitment for final analysis.

Supplementary Table S1 Sex differences in the reference sites of intracranial arteries.
