# Supplementary figures and images for: Sex Differences in Intracranial Atherosclerotic Plaques Among Patients With Ischemic Stroke
Source: Front Cardiovasc Med. 2022 Jun 30;9:860675. doi: 10.3389/fcvm.2022.860675 (PMC9280275; doi:10.3389/fcvm.2022.860675)

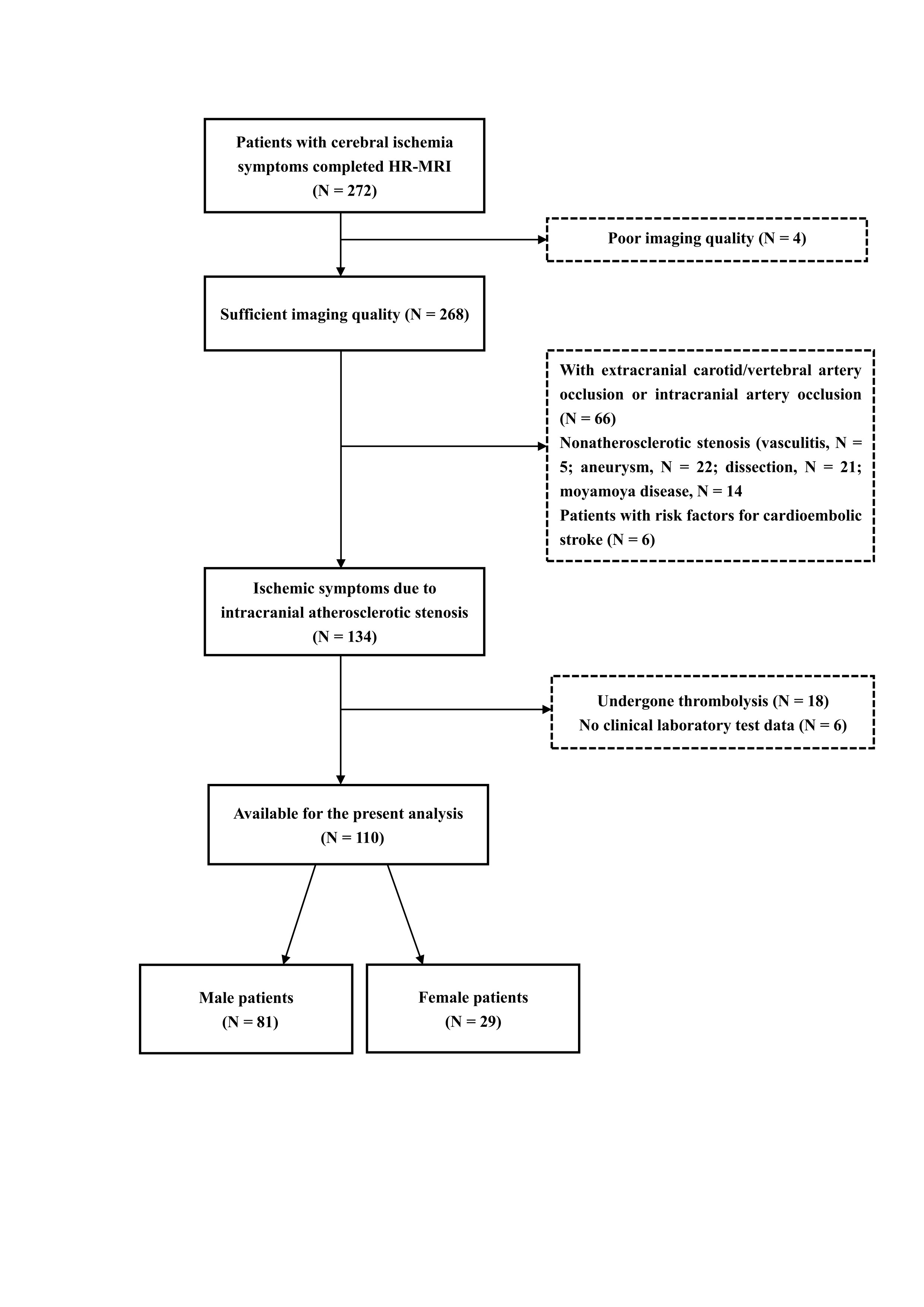

Supplement: Supplementary Figure S1 — Flow chart of patient recruitment for final analysis. [file Image_1.TIF]
