# Supplementary material for: Sex Differences in Intracranial Atherosclerotic Plaques Among Patients With Ischemic Stroke
Source: Front Cardiovasc Med. 2022 Jun 30;9:860675. doi: 10.3389/fcvm.2022.860675 (PMC9280275; doi:10.3389/fcvm.2022.860675)
Supplement: Supplementary Table S1 — Sex differences in the reference sites of intracranial arteries. [file Table_1.DOCX]

Supplementary Material

**Table S1**. Sex differences in the reference sites of intracranial arteries

|  | All (n = 311) | | | Culprit plaque (n = 97) | | | Uncertain plaque (n = 65) | | | Non-culprit plaque (n = 150) | | |
| --- | --- | --- | --- | --- | --- | --- | --- | --- | --- | --- | --- | --- |
| Characteristics | Male | Female | *P* | Male | Female | *P* | Male | Female | *P* | Male | Female | *P* |
|  | (n = 236) | (n = 75) |  | (n = 71) | (n = 26) |  | (n = 52) | (n = 12) |  | (n = 113) | (n = 37) |  |
| Outer wall area (mm^2^) | 15.89 (11.9, 20.9) | 13.14 ± 4.53 | 0.001 | 14.52 ± 4.59 | 10.51 ± 3.75 | 0.001 | 16.1 (11.4, 20.8) | 15.9 ± 4.78 | 0.783 | 18.2 ± 6.41 | 14.07 ± 4.10 | 0.001 |
| Lumen area (mm^2^) | 6.6 (4.8, 9.3) | 5.5 (4.3, 7.4) | 0.003 | 5.94 ± 2.10 | 5.22 ± 1.94 | 0.128 | 6.2 (4.0, 8.5) | 7.24 ± 2.99 | 0.536 | 7.8 (5.72, 10.9) | 5.5 (4.4, 7.6) | 0.001 |
| Wall area (mm^2^) | 9.0 (7.0, 11.6) | 7.14 ± 2.89 | 0.001 | 8.58 ± 3.12 | 5.29 ± 2.43 | 0.001 | 10.1 ± 3.88 | 8.71 ± 2.41 | 0.236 | 9.59 ± 3.49 | 7.94 ± 2.72 | 0.004 |
